# Enterococci from Raw-Milk Cheeses: Current Knowledge on Safety, Technological, and Probiotic Concerns

**DOI:** 10.3390/foods10112753

**Published:** 2021-11-10

**Authors:** Amarela Terzić-Vidojević, Katarina Veljović, Nikola Popović, Maja Tolinački, Nataša Golić

**Affiliations:** Institute of Molecular Genetics and Genetic Engineering, University of Belgrade, Vojvode Stepe 444a, 11042 Belgrade, Serbia; katarinav@imgge.bg.ac.rs (K.V.); popovicnikola@imgge.bg.ac.rs (N.P.); maja_tolinacki@imgge.bg.ac.rs (M.T.); natasag@imgge.bg.ac.rs (N.G.)

**Keywords:** *Enterococcus* spp., raw-milk cheeses, safety, technological characteristics, probiotic properties

## Abstract

The present study is focused on the safety, technological characteristics, and probiotic evaluation of *Enterococcus* species from different artisanal raw milk dairy products, mainly cheeses with ripening. Apart from proteolytic and lipolytic activities, most enterococci show the ability to metabolize citrate and convert it to various aromatic compounds. Long-ripened cheeses therefore have a specific flavor that makes them different from cheeses produced from thermally treated milk with commercial starter cultures. In addition, enterococci are producers of bacteriocins effective against spoilage and pathogenic bacteria, so they can be used as food preservatives. However, the use of enterococci in the dairy industry should be approached with caution. Although originating from food, enterococci strains may carry various virulence factors and antibiotic-resistance genes and can have many adverse effects on human health. Still, despite their controversial status, the use of enterococci in the food industry is not strictly regulated since the existence of these so-called desirable and undesirable traits in enterococci is a strain-dependent characteristic. To be specific, the results of many studies showed that there are some enterococci strains that are safe for use as starter cultures or as probiotics since they do not carry virulence factors and antibiotic-resistance genes. These strains even exhibit strong health-promoting effects such as stimulation of the immune response, anti-inflammatory activity, hypocholesterolemic action, and usefulness in prevention/treatment of some diseases.

## 1. Introduction

The genus *Enterococcus* is one of the main genera of the lactic acid bacteria (LAB) group, with over 50 species and subspecies [1]. Enterococci belong to the family *Enterococcaceae*. They are gram-positive, catalase- and oxidase-negative, non-spore-forming, facultative anaerobic cocci occurring either as single bacteria, in pairs, in short chains, or in groups [2,3]. They are found in a variety of habitats, such as the gastrointestinal tract of humans and animals and plants, soil, water, and foods of animal origin, especially cheeses and fermented sausages [4,5,6,7,8,9,10,11]. One of the reasons for their prevalence in diverse niches is their robust nature since the majority of them have the ability to grow at temperatures from 10 to 45 °C, in 6.5% NaCl, 40% bile, and pH from 4 to 9.6. Additionally, they can survive heating at 60 °C for 30 min [2,3].

In Southern European countries, such as Portugal, Spain, Italy, and Greece, enterococci are highly valued and used in cheese production as components of mixed starter cultures for development of taste and flavor during cheese ripening, probably through proteolysis, lipolysis, and citrate breakdown [3]. In addition, certain enterococci have the ability to produce bacteriocins active against relevant spoilage-causing and pathogenic microorganisms in foods (such as *Listeria monocytogenes*) and possess appropriate probiotic properties, which are strong arguments for their application in the production of fermented food [12,13,14,15]. The total viable count of enterococci in Mediterranean-type cheese curds is in the range between 10^4^ and 10^6^ CFU/g at the beginning of ripening and between 10^5^ and 10^7^ CFU/g at the end of cheese ripening, thereby contributing to their typical sensory properties [16].

On the other hand, Northern European countries have considered enterococci undesirable in the food industry because there is an opinion that enterococci, inasmuch as they are predominant in the gastrointestinal tract of humans and animals, are indicators of fecal pollution (enterococci were formerly known as the “fecal streptococci” or “Lancefield’s group D streptococci”), transmitting antibiotic-resistance genes and virulence factors [17,18]. However, the presence of enterococci in foods does not always have to be associated with fecal contamination [19], as they can enter food from other sources, such as water, animal feed, or the animal’s exterior [17]. The European Union (EU) established a maximum level for the presence of coliforms and *Escherichia coli*, both considered as indicators of hygiene, while no limit was set for enterococci [20]. Moreover, results obtained by Birollo et al. [21] showed that enterococci had little value as hygiene indicators in industrially produced foods. In accordance with these results, it is obvious that enterococci are ubiquitous in the environment and can be present in food without being of fecal origin [22].

Although enterococci exhibit useful benefits for the production of fermented food, they are also identified as opportunistic pathogens that can cause various human diseases, such as bacteremia, urinary tract infections, and endocarditis [23]. Two enterococci species are suggested as responsible for these infections—*Enterococcus faecalis* and *Enterococcus faecium* [24,25]. They show a high level of virulence and transmission of antibiotic resistance genes, particularly the vancomycin-resistant ones [26,27]. Despite recent knowledge that the pathogenesis of enterococci is a strain-dependent trait and is more common to clinical enterococci than to enterococci from food [28,29], *Enterococcus* species do not have a generally recognized as safe (GRAS) status [30], nor have they been included on the qualified presumption of safety (QPS) list [31,32]. In connection with the above, a previous study of ours [33] threw light on the diversity, antibiotic susceptibility, virulence traits, production of biogenic amines, and technological properties of 636 enterococci isolated from 55 artisanal dairy products. The results showed that only 0.8% of them satisfy safety criteria for use in the dairy industry and indicated that, although isolated from food, enterococci can be reservoirs of antibiotic-resistance and virulence genes as well as producers of biogenic amines, thereby stressing that detailed testing of each individual enterococcal strain is necessary before its potential use in the food industry [17].

Artisanal dairy products are mainly produced from raw milk, which is rich in a variety of autochthonous microorganisms that determine their typical aroma. Studies on the microbiota of traditional cheeses of Mediterranean and Western Balkan Countries produced mainly from raw milk of sheep, goats, or cows indicate that enterococci are a relevant component of the non-starter LAB. Findings obtained by LAB identification showed that about one-third of all isolated LAB are *Enterococcus* species [34,35,36,37,38,39,40]. Additionally, similar results were obtained by analyzing bacteria from traditional Brazilian cheeses [41]. Apart from dairy products, 20% of total bacteria isolated from raw cow’s milk belonged to *Enterococcus* species [42]. *Enterococcus faecium*, *Enterococcus faecalis,* and *Enterococcus durans* are found as the most prevalent species of enterococci in raw-milk cheeses [3,11,18,43,44,45,46,47,48,49,50,51,52,53,54]. In addition, *Enterococcus italicus*, *Enterococcus galinarum*, *Enterococcus avium*, *Enterococcus casseliflavus*, *Enterococcus lactis*, *Enterococcus hermanniensis*, and *Enterococcus gilvus* were isolated in a low percentage from sweet kajmaks, Turkish white cheese, artisanal Italian cheeses (the Toma Piemontese and Vastedda della valle Belìce cheeses), 60-day-old raw milk Zlatar cheese, artisanal Istrian raw-milk cheese, and Ezine nonstarter long-ripened white cheese, respectively [39,41,55,56,57,58,59,60]. Morandi et al. [61] described *Enterococcus lactis* sp. nov., a new species found during isolation of the autochthonous microflora of an Italian raw-milk cheese (Bitto).

**Figure 1:**
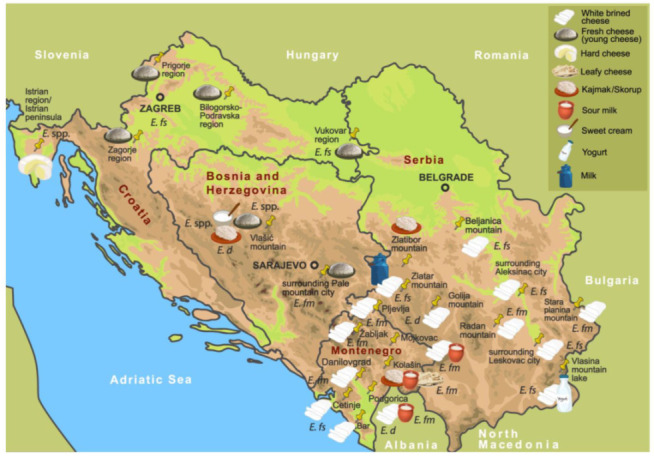
Artisanal raw milk dairy products manufactured in the region of Western Balkan Countries as a source of *Enterococcus* spp. Note: Most prevalent *Enterococcus* spp. in certain geographical localities: *E*. spp., *Enterococcus* species; *E*. *fm*, *Enterococcus faecium*; *E*. *fs*, *Enterococcus faecalis*; *E*. *d*, *Enterococcus durans* [11,54].

Extensive study of enterococcal diversity in the Western Balkans region showed that enterococci are present in various types of autochthonous dairy products, such as white brined cheeses and salted kajmaks in Serbia; hard and soft fresh cheeses in Croatia; young cheeses, sweet creams, and sweet kajmaks in Bosnia and Herzegovina; and leafy, white and semi-hard cheeses and skorups in Montenegro (Figure 1) [11,33,47,48,52,53,62,63]. Most of them were identified as *E. durans* (44%), *E. faecalis* (35.9%), and *E. faecium* (17.9%), while less than 2% were *E. italicus* strains [33]. Similar data were reported by authors who determined the enterococcal population in artisanal dairy products manufactured in other regions [64,65].

The aim of the present work is to point out advantages and disadvantages of enterococci isolated from raw-milk cheeses and summarize current knowledge about their safety, technological properties, and probiotic capacities on the one hand and stress their great potential for application in the food industry on the other, considering that some of these properties can lead to serious health problems in immunocompromised patients.

## 2. Safety of Using *Enterococcus* spp. in Dairy Food

According to the European Food Safety Authority (EFSA), members of the genus *Enterococcus* are not recommended for the QPS list [31] nor for GRAS status according to the Federal Food, Drug, and Cosmetic Act, USA [30], due to their controversial epidemiological status. In regard to their safety status, enterococci are assigned to risk group 2, together with other microorganisms carrying virulence factors [66]. The safety of dairy enterococci will be discussed here, together with recommended methods for its evaluation and the urgent need for new proposals and legislation to determine the safety of enterococci used in dairy and pharmaceutical industry.

On the one hand, enterococci have been associated with bacteremia and nosocomial infections mostly related to the presence of intrinsic or acquired antibiotic-resistance genes located on chromosomes, plasmids, or transposons [23,67] as well as to that of genes encoding virulence factors [68]. Moreover, a controversial correlation between *E faecalis* and pancreatic and colorectal cancers was recently suggested [69,70]. Besides the ability to cause diseases, the presence of resistance and virulence genes represents a huge threat to global health due to the possibility of horizontal transfer of these genes through the food chain to clinically relevant pathogens [71]. It is noteworthy in particular that vancomycin-resistant enterococci (VRE) represent a substantial problem in healthcare since vancomycin is often used as the last alternative in the treatment of nosocomial infections caused by multiple antibiotic-resistant enterococci [30]. However, while the horizontal transfer of resistance genes from *E.faecalis* to methicillin-resistant *Staphylococcus aureus* (MRSA) was reported [72,73], the results of Faron et al. [74] indicated that this horizontal transfer occurs at a very low frequency.

Regarding the presence of virulence genes, previous results of ours indicated that the genes encoding gelatinase (*gelE*), cytolysin activator (*cylA*), hyaluronidase (*hyl_Efm_*), aggregation substance (*agg*), collagen adhesin (*ace*), and enterococcal surface protein (*esp*) are sporadically detected in dairy isolates [68]. It should be emphasized that there are two groups of virulence factors: surface factors involved in the colonization of host cells and factors causing damage to the host’s tissues [75]. The virulence factors enabling host colonization, including aggregation substance (AS), cell wall adhesin (EfaA), collagen-binding protein (Ace), and enterococcal surface protein (Esp), provide for binding of enterococci cells to receptors on the host’s epithelium [16]. Hence, although colonization represents the first step in pathogenesis, adhesion factors alone are not necessarily involved in pathogenicity. The genes for hemolytic activity as well as for adhesions (*esp* and *efaA*) were found with high frequency in all tested *E. faecalis* strains [24]. Although the results of Popović et al. [68] indicated the *agg* and *esp* genes to be positively associated with probiotic features in dairy isolates of enterococci, this must unquestionably be taken with precaution since AS is part of the conjugation process responsible for exchange of genetic material between bacterial cells during the conjugative transfer of sex pheromone plasmids and could favor the horizontal transfer of antibiotic-resistance genes [76]. In addition, the *esp* gene encoding the Esp protein is located on the pathogenicity island (PAI), which also contains genes involved in antibiotic outflow [77]. Moreover, the Esp protein is involved in adhesion, colonization, and evasion of the immune system as well as in biofilm formation, an important feature in horizontal gene transfer and the occurrence of antibiotic resistance [78].

In order to use a particular strain as a food supplement, it is necessary to evaluate the presence/absence of transferable antibiotic resistance and/or virulence genes since the presence of genes encoding antibiotic resistance and virulence factors in enterococcal strains intended for starter culture in food production could have substantial effects on human and animal health due to potential horizontal gene transfer throughout the food chain or even cause the occurrence of disease [79].

The safety status of 636 dairy isolates belonging to the species *E. durans*, *E. faecium*, *E. faecalis*, *E. italicus,* and *Enterococcus hirae* was investigated previously using various microbiological and molecular methods [24,33,68]. Interestingly, while results obtained by the disk diffusion method recommended by the Clinical and Laboratory Standards Institute (CLSI) [37] revealed that 29.1% enterococci were antibiotic-susceptible, use of the microdilution method showed that among them even 57% were resistant to ciprofloxacin or gentamicin, indicating that the method used for antibiotic susceptibility testing should be carefully chosen [68]. The high frequency of antibiotic resistance in the tested dairy enterococcal isolates was in accordance with published data on the results of extensive use of antibiotics in livestock production [18,23,71,75]. The further spread of antibiotic-resistance genes in other food-associated bacteria could be the cause of disappearance of natural sources of LAB suitable for the dairy industry.

In the case of functional foods, the EFSA recommendations on strain safety should be followed [80]. According to those recommendations, enterococcal isolates from food must be considered individually, and the risks to health must be excluded for each particular strain. To be specific, strains that do not carry the *IS16* marker, exclusively detected in clinical isolates of *E.faecium* [81] as well as the *hyl_Efm_* and *esp* genes, can be regarded as safe [82]. Previously published data related to enterococci isolated from different environments indicated that virulence factors are more prevalent in *E. faecalis* isolates than in *E. faecium* strains [83,84].

Previous research designed to evaluate the safety status of dairy enterococci originating from artisanal dairy products from the Western Balkans showed that virulence genes were sporadically present in the analyzed isolates [68]. While the *esp* gene was previously detected in isolates originating from water, vegetables, and raw milk [85], the study of Popović et al. [68] for the first time revealed the presence of the *esp* gene in dairy isolates mostly associated with their adhesion properties, thereby pointing to its role in gut colonization rather than to virulence features. To be specific, the *esp* gene in dairy enterococci was associated to a greater extent with the *agg*, *efaA_fs_*, and *efaA_fm_* genes, which have a role in adhesion and colonization [68]. Although *E. durans* strains have been characterized as good candidates for use in the food and pharmaceutical industries, all of them had some of the tested virulence genes [68], suggesting that *E. durans* should be checked more rigorously. It is important to highlight that genes encoding degradative enzymes associated with tissue damage were not detected, with the exception of *gelE* [68]. To judge from published data, the *gelE* gene could be silent [83], a hoped-for possibility. Interestingly, only five isolates out of 636 tested enterococci strains were completely free of virulence factor genes [33]. In addition, 30 out of 75 analyzed strains were able to form a biofilm [68]. Published data suggest that biofilm formation is a common trait of commensal enterococci isolates from human feces, indicating that this trait is not necessarily involved in pathogenicity but could be associated with adhesion and colonization properties [86,87].

## 3. Technological Characteristics of Enterococci

Natural LAB isolates possess a system of catabolic enzymes for proteolysis, lipolysis, and citrate metabolism that is better adapted to the cheese environment than the one in commercial starter cultures [88]. Enterococci are highly important in traditional fermented foods, particularly in artisanal cheeses, due to their technological properties, viz., different activities (acidifying, proteolytic, and lipolytic), citrate utilization, and production of aromatic volatile compounds that provide the specific sensory characteristics of many cheese varieties [1,3]. Due to their desirable metabolic properties, it has been suggested that certain enterococci strains could be used as part of existing starter cultures in the production of various cheeses [3,30], such as Bitto [61], water-buffalo Mozzarella [89], feta [90], Venaco [91], Cebreiro [92], cheddar [93], Koopeh [94], Tulum [95], and Lighvan [96], known to consumers around the world.

### 3.1. Acidification Activity

The main characteristic of LAB is production of lactic acid by fermentation of lactose. In this way, various effects are achieved: (a) decrease of milk pH and coagulation of casein; (b) increase in acidity of the environment due to lowering of the milk’s pH value, which makes possible control of the growth of pathogenic and spoilage bacteria; (c) positive action of casein coagulation on the rheological properties of dairy products; and (d) determination of the final flavor quality of ripened cheeses as a result of acidification [11]. However, not all LAB have the ability to acidify milk. Some of them lower milk pH rapidly, some do so slowly, and some LAB do not acidify milk. Enterococci belong to the group of LAB, which in general exhibit low or medium milk-acidifying ability [22]. Numerous published data confirm the poor acidifying capacity of these bacteria in milk showing a pH below 5.0 after 24 h of incubation at 30–37 °C [97,98,99,100,101,102]. Examining 636 enterococcal strains isolated from various types of dairy products, investigators found only 27 isolates (4.2%) possessing the ability to form curd after 6 h of incubation at 37 °C [33]. Since starter cultures are defined as isolates, which produce sufficient acid to reduce the pH of milk to 5.3 in 6 h at 30–37 °C [103], enterococci with low acidifying ability would not be good candidates for starter cultures in cheese manufacture, but they could be useful as adjunct cultures in combination with high-capacity acidifiers due to exhibiting other technological characteristics that are desirable [100].

However, some authors reported good acidification ability of enterococci. Thus, for example, Ribeiro et al. [104] found six *E. faecalis* fast-acidifier strains that lowered the pH of UHT milk from a starting value of 6.48 to 5.13–4.87 over the course of 6 h at 30 °C. *Enterococcus faecalis* strain SLT13 reduced the milk´s pH to 4.29 after 18 h of growth at 37 °C [105], while two *Enterococcus* sp. isolates from Kashar cheese lowered it to 4.08 for 24 h [106]. According to results reported by de Paula et al. [107], better acidification values can be achieved by prolonging cultivation time. A few authors reported that *E. faecalis* strains reduce milk pH faster than *E. faecium* and *E. durans* strains [22,108]. In contrast, *E. faecium* strains from Turkish Tulum cheese showed faster fermentation activity than *E. faecalis* [109], while Jaouani et al. [110] did not find differences in the rate of acidification of milk between *E. faecalis* and *E. faecium* strains after 24 h.

In view of these contradictory findings, it is apparent that further research is needed in the future to clarify which *Enterococcus* species has the better acidifying ability or if this property is strain-specific.

### 3.2. Proteolytic Activity

The degradation of casein due to proteolytic and peptidolytic activities plays a major role in development of the texture and organoleptic properties of cheese [3,105]. The positive role of enterococci in cheese production is associated with their proteolytic activity [111]. Apart from the ability of enterococci to grow in an environment with a wide range of temperatures, high salt content, and low pH values, the predominance of enterococci in cheeses with a long ripening period [34,58,59] is made possible by their production of proteolytic enzymes, which provides them with the peptides and amino acids essential for their growth [111]. However, despite the fact that enterococci were found to be the predominant LAB group in raw milk dairy products [11], there are only a few studies treating the proteolytic system of enterococci in comparison with *Lactobacillus* and *Lactococcus* species [112,113]. The proteolytic enzymes of enterococci have been insufficiently examined, and this may be one of the reasons for the limited use of enterococci in the production of traditional cheeses at the industrial level.

Specific for the proteolytic system of enterococci is the fact that it is characterized by the presence of gelatinase, an extracellular zinc metalloprotease capable of hydrolyzing gelatin, elastin, collagen, and hemoglobin [1]. Gútiez et al. [114] showed that gelatinase present in *E. faecalis* isolates originating from food and the ambient environment was responsible for casein degradation and formation of bioactive peptides, a circumstance that can affect human health. Gelatinase is encoded by the *gelE* gene as a virulence factor, which plays a significant role in the pathogenicity of enterococcal strains [115]. However, *gelE* alone was not proven to be directly responsible for infection [116], nor was it established that the presence of functional gelatinase is associated with all virulence features since it is not required for enterococci to cause disease [117]. Based on these findings, it can be asserted that proteolytic enterococci from food containing gelatinase [115,117] does not have to be excluded from eventual commercial use in the food industry [118,119].

While some authors have reported high levels of proteolytic activity [98,109,120], others considered that enterococci have weak proteolytic activity [97,99]. The data in the literature are very diverse. For instance, only one of seven tested *E. faecalis* strains from an artisanal Pico cheese showed low proteolytic activity [104]. Twenty-one *E. faecalis* isolates from artisanal raw milk Zlatar cheese degraded casein poorly, but three *E. faecalis* strains completely degraded α_s1_- and κ-casein after 3 h of incubation and β-casein after 30 min of incubation, indicating that proteolytic activity is a strain-dependent property [35].

In line with the fact that gelatinase is more frequently detected in *E. faecalis* than in *E. faecium* [121,122], many studies reported that *E. faecalis* showed better proteolytic activity than other *Enterococcus* species [99,108,123]. Terzic-Vidojevic et al. [33] found that 111 (17.5%) out of 636 examined enterococci degraded β-casein and that the number of *E. faecium* and *E. faecalis* strains was approximately equal. On the other hand, Mrkonjic Fuka et al. [59], Dagdemir [124], and Cosentino et al. [125] obtained results indicating better caseinolytic activity in the species *E. faecium*. Interestingly, according to the observation of some authors [126,127], enterococci show higher proteolytic activities than other LAB, a fact that increases their significance for use in cheese production.

A deeper evaluation of the proteolytic activity of enterococci is needed in view of conflicting scientific knowledge about it, specific biochemical properties of the genus, and its potentially great importance for further use in the dairy industry as part of mixed starter cultures.

### 3.3. Lipolytic Activity

Enterococci are one of many bacterial groups that show lipolytic and esterolytic activity, producing both lipases and esterases, which hydrolyze triglycerides to free fatty acids, glycerol, and intermediates, such as mono- and diglycerides [128,129]. The lipolytic and esterolytic system of enterococci is therefore very useful in food fermentation, especially in the case of dairy and meat products that require ripening [10,22,128,130].

Contradictory data regarding the lipolytic activities of enterococci have been reported to date, the obtained results ranging from pronounced lipolytic activity [98], to relatively high lipolytic activity [99], to low lipolytic activity, or its absence [89,104,108,110]. Carrasco de Mendoza et al. [131] concluded that the lipolytic activity of enterococci in milk is strain dependent. Morandi et al. [132] found that *E. faecium* strains from dairy products in northwestern Italy were the most lipolytic, followed by the tested *E. faecalis* and *E. durans* strains. On the other hand, lipolytic ability was confirmed in one-third of enterococci isolated from an artisanal Istrian raw-milk cheese [59]. *Enterococcus faecalis* showed better lipolytic activity than *E. faecium* and *E. durans* [59,109]. Regardless of differences existing between *Enterococcus* species as well as between strains within the same species, strains with high lipolytic characteristics should be subjected to further examination as a potential commercial adjunct culture in production of fermented food.

### 3.4. Production of Aromatic Compounds

The bulk of aromatic compounds in cheese ripening is produced during citrate metabolism by the activity of LAB, the majority of which are often enterococci. Results obtained by Terzic-Vidojevic et al. [33] showed that 40.9% tested enterococci from various artisanal dairy products utilized citrate as the only source of carbon. During manufacturing and ripening of raw-milk cheeses, citrate can be degraded over different metabolic pathways, giving significant amounts of various aromatic compounds (mainly acetate, acetaldehyde, acetoin, and diacetyl) that are responsible for the specific and intense flavor of final raw-milk cheese compared to that of cheeses made with pasteurized milk [133]. The most significant contribution to the buttery and ‘‘buttermilk” aroma and flavor of dairy products was made by diacetyl, which is a volatile compound formed as an end product during the conversion of citrate to pyruvate [134]. The appearance of certain aromatic compounds in cheese is dependent on many factors that influence citrate metabolism, such as the type of LAB, cell density, culture condition, environment pH, and lactate concentration [135,136].

During the last several decades, the citrate metabolism of enterococci has been examined by a number of investigators. The obtained results showed that enterococci are better diacetyl-acetoin producers than other LAB [99,137]. It was established that enterococci produce numerous volatile compounds and contribute to the formation of the cheese aroma, especially during ripening [17,91,100,138]. In the study of Cárdenas et al. [139], a total of 41 volatile compounds were identified in experimental cheeses manufactured with *E. faecium* CECT 8849.

Not all LAB have the capacity to metabolize citrate [129]. Significant differences among *Enterococcus* species and strains were found with respect to diacetyl and acetoin production. The highest production of diacetyl was obtained with the strain *E. faecium* C1W5, followed by the strains *E. faecalis* N8W4 and N0W5 [140]. On the other hand, several authors reported that certain *E. durans* strains had better production of diacetyl compared to other *Enterococcus* species [33,101,125,141]. All 56 tested *E. faecalis* strains completely utilized both citrate and pyruvate after 16 h [99], and six out of seven *E. faecalis* strains from Pico cheese produced diacetyl, among which four strains showed medium and two strains low production of diacetyl [104]. In addition, it was shown that *E. faecalis* strains were better producers of acetoin than other enterococci strains, since 157 of 229 *E. faecalis* strains gave a positive reaction for acetoin production in contrast to 138 of 280 *E. durans* and 48 of 114 *E. faecium* strains [33].

To judge from previously published data, we can conclude that citrate utilization by enterococci is an important technological characteristic and that enterococci, as a predominant part of non-starter lactic acid bacteria of raw milk products, determine their specific flavor. However, various data can be found regarding the abilities of different enterococci species and strains to convert citrate and pyruvate to aromatic compounds, so it is obvious that citrate metabolism is a strain-specific property.

## 4. Probiotic Potential of *Enterococcus* spp.

Enterococci have been traditionally thought to be indicators of fecal contamination as well as a cause of nosocomial infections and food spoilage. For that reason, their safety status is still controversial, as was discussed above. However, many enterococcal dairy isolates have probiotic effects, thereby contributing positively to human and animal health. In spite of safety concerns and due to the lack of legislation, some of them have already been used in commercial probiotic products, such as Cylactins (Hoffmann-La Roche, Basel, Switzerland), Fargo 688s (Quest International, Naarden, Netherlands), ECOFLOR (Walthers Health Care, DenHaag, Netherlands), Symbioflor 1 (SymbioPharm, Herborn, Germany), and Cernivet^®^ and FortiFlora^®^ (containing *E. faecium* SF68^®^) (Cerbios-Pharma SA, Barbengo-Lugano, Switzerland) [142]. Notably, Symbioflor 1 (containing *E. faecalis* DSM 16431) has a history of long-term safe use, and its safety was proven by whole-genome sequencing (WGS) [142]. In recent years, WGS data have been increasingly used to identify potential probiotic strains as well as to characterize strains in terms of their potential functionality for health [80].

Probiotics have been defined as “live microorganisms that confer health benefits to the host when ingested in adequate amounts” [143,144]. Enterococci are generally widespread in nature due to their ability to survive harsh conditions, making them good probiotic candidates. One of the important characteristics of probiotics is the ability to survive the conditions of gastrointestinal tract [143,145]. Popović et al. [15] reported that 13 *E. durans* strains from traditional cheeses of the Western Balkan Countries showed good probiotic properties, such as surviving simulated gastric conditions and prolonged exposure to bile salts and pancreatic enzymes, pointing to their gut commensal origin.

Another important probiotic characteristic is antimicrobial activity. Enterococcal bacteriocins, so-called enterocins, mostly belong to Class-II bacteriocins [146]. Many enterococci simultaneously synthesize several bacteriocins active against a number of pathogens and could be good candidates for use as antibiotic replacements or food preservatives [32,147]. Numerous literature data report a strong antimicrobial action of certain *E. durans*, *E. faecium*, and *E. faecalis* strains against one or more pathogenic bacteria, indicating that natural dairy enterococcal isolates produce enterocins with a broad spectrum of activity [14,15,19,68,148,149,150,151].

One of the criteria for selection of probiotics could be adhesion ability to intestinal epithelial cells (IEC), a prerequisite for gut colonization and persistence as well as for competitive exclusion of pathogens [143,152]. However, the cell-surface proteins involved in colonization of enterococci are for the most part virulence factors, as noted above [87]. Adhesion to mucin and IEC of 13 *E. durans* dairy isolates originating from artisanal dairy products was reported previously [68]. Although strain-specific differences were noticed, all 13 isolates showed a high adhesion potential. Most of them harbor the *efaA_fs_*, *efaA_fm_*, *agg,* and *esp* genes associated with adhesion ability. Interestingly, the *esp* gene was detected in even as many as six out of 13 tested strains. Although according to EFSA [82], occurrence of the *esp* gene is an undesirable property in probiotic enterococcal strains, the study of Popović et al. [68] revealed that it was mostly correlated with genes having a role in gut colonization, viz., the *agg*, *efaA_fs_*, and *efaA_fm_* genes, while virulence factors important for tissue damage were not detected [68]. However, in two *E. durans* strains, detection of the *esp* gene was related to the ability to form a biofilm, a virulence factor important in enterococcal pathogenicity [68,153]. In addition, the ability to counteract the negative effects of pathogens by competitive exclusion is a highly desirable property that should be taken into account in the selection of probiotic bacteria. The study of Popović et al. [68] revealed that enterococcal dairy isolates were able to reduce adhesions of *Escherichia coli* ATCC 25,922 and *Salmonella* Enteritidis 654/7E to HT29-MTX. Similarly, Jin et al. [154] reported that adhesion of enterococci to IEC limits the excessive pathogen growth.

In view of all the above-mentioned controversial features of enterococci, it would appear that one of the safe options in seeking to exploit their health-promoting properties and avoid the risk of their potential virulence as well as the danger of horizontal transfer of genes encoding virulence factors and resistance to antibiotics is to use them as postbiotics, non-viable bacterial extracts, and metabolic by-products. The effectiveness of heat-killed *E. faecium* BGPAS1-3 as a potential postbiotic was reported by Popović et al. [15]. To be specific, the heat-killed BGPAS1-3 postbiotic exhibited the same strong anti-listerial effect inhibiting the adhesion of *L. monocytogenes* ATCC 19111 to differentiated Caco-2 IEC as live bacteria.

One of the most critical steps in pathogenesis is invasion by the pathogens and their passage through the selectively permeable intestinal epithelium barrier, a multi-protein complex between adjacent epithelial cells composed of what have been denoted as tight-junction proteins [155]. In particular, *L. monocytogenes* is one of the pathogens that can disrupt tight-junction transmembrane structures, thereby causing epithelial barrier dysfunction [156]. Interestingly, the heat-killed BGPAS1-3 postbiotic was able to prevent tight-junction disruption in the differentiated Caco-2 monolayer after infection by *L. monocytogenes* ATCC 19111 through stimulation of the expression of claudin and occludin, important tight-junction proteins in Caco-2 cells, suggesting that enterococci could be good regulators of the epithelial barrier´s function [15], particularly as a safe postbiotic and controllable therapeutic. Similarly, *E. faecium* NCIMB 10415 was shown to improve the intestinal barrier´s integrity [157].

Moreover, IEC were the place where the presence of pathogens was first recognized by pathogen-associated molecular patterns (PAMPs) with pathogen recognition receptors (PRRs), including toll-like receptors (TLRs), upon which IEC produce antimicrobial molecules and activate the innate immune response and stimulate the production of protective cytokines (such as IL-8) and that of transforming growth factor (TGF)-β [158,159,160,161,162]. IL-8 is secreted by IEC as well as by several other cell types and has an important role in the activation of leukocytes, initiating the acute inflammatory response in listeriosis [156]. On the other hand, TGF-β prevents inflammation-mediated epithelial damage, thereby protecting the epithelial barrier´s integrity [163]. Popović et al. [15] reported that the *E. faecium* BGPAS1-3 heat-killed postbiotic, besides having an antimicrobial anti-listerial effect, exhibits immunomodulatory activity through stimulation of the production of protective IL-8 and TGF-β in IEC as well as through modulation of MyD88-dependent TLR2 and TLR4 pathways. The findings are in accordance to other published data indicating that enterococci could be used as immunomodulators [164,165]. It is concluded that manipulation of TLR expression can be the way enterococci achieve immunomodulatory activity [166].

The immunomodulatory effect of enterococci can also be related to the production of short-chain fatty acids, particularly butyrate, such as in the case of *E. durans* M4-5 [167]. Moreover, enterococci exhibit an anticarcinogenic and hypocholesterolemic effect, e.g., *E. faecium* M74^®^ and *E*. *durans* KLDS 6.0930 were shown to reduce the level of cholesterol in serum [168]. The importance of enterococcal probiotic strains has been confirmed not only in animals but also in humans, for example, the assessment of the effectiveness of *E. faecium* SF68^®^ and *E. faecalis* Symbioflor 1 in humans for the treatment of antibiotics-associated diarrhea [169].

**Figure 2:**
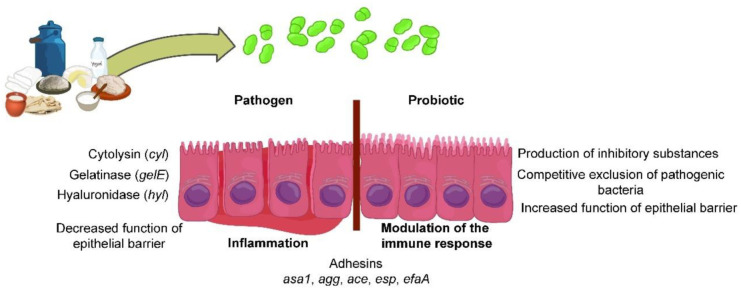
Contrasting effects of enterococci-from emergence of pathogens to potential probiotic action.

Safety and probiotic aspects of enterococci are summarized in Figure 2. Enterococci isolated from fermented dairy products show both a pathogenic and a probiotic potential. Their pathogenic potential is manifested in the synthesis of enzymes (cytolysin, gelatinase, hyaluronidase) that can degrade various proteins as well as whole cells, lower effectiveness of the epithelial barrier, and lead to inflammation. On the other hand, enterococci can have a probiotic effect that is realized through various mechanisms (synthesis of antimicrobial molecules, competitive exclusion of pathogens). Soluble and cell-bound molecules can enhance the epithelial barrier´s function and modulate the immune response. Various adhesins expressed on the cell surface (*asa1*, *agg*, *ace*, *esp*, *efaA*) play a role in cell binding to the host and colonization.

## 5. Conclusions

As members of the LAB group, enterococci are well adapted for survival and persistence in various ecological niches. This review provides an update of information about *Enterococcus* species originating from raw-milk cheeses and their safety, technological characteristics, and probiotic capacity. It is known that enterococci are bacteria with “two faces” since they show desirable technological characteristics and probiotic properties but at the same time carry a number of virulence factors that make them undesirable for application in the food industry. The article presents numerous published data from which it can be concluded that there are no species of enterococci exclusively safe or exclusively unsafe for human health. All their properties, good or bad, are strain-specific.

Large numbers of in-vitro and in-vivo tests are needed to guarantee that a given *Enterococcus* strain is quite safe and suitable as a probiotic strain for potential application in the production of fermented foods. Modern techniques of molecular biology can help to obtain this knowledge and make it possible to develop improved legal standards and guidelines to ensure the faster introduction of enterococci for commercial purposes.

## Data Availability

The data presented in this study are available on request from the corresponding author.
